# Clinical outcomes of flap reconstruction following lower extremity soft tissue sarcoma resection: a single-center retrospective experience

**DOI:** 10.3389/fsurg.2026.1906688

**Published:** 2026-09-03

**Authors:** Xinxin Guo, Jianpeng Li, Jingyu Zhang, Yongcheng Hu

**Affiliations:** 1 Clinical School/College of Orthopedics, Tianjin Medical University, Tianjin, China; 2 Department of Plastic Surgery, Tianjin Hospital, Tianjin, China; 3 Department of Orthopedics, Tianjin Fifth Central Hospital, Tianjin, China; 4 Department of Bone Tumor and Soft Tissue Oncology, Tianjin Hospital, Tianjin, China

**Keywords:** adjuvant therapy, flap reconstruction, lower extremity, soft tissue sarcoma, limb salvage

## Abstract

**Background:**

Soft tissue sarcomas (STSs) represent a diverse category of tumors with a propensity for the lower limbs. Wide surgical resection is the primary therapeutic approach, often requiring immediate flap reconstruction with or without perioperative adjuvant therapy. This study aims to share our clinical experience and assess the efficacy of customized flap reconstruction designed according to the precise defect area after lower extremity STS resection.

**Methods:**

This retrospective analysis reviewed 15 cases of lower extremity STS with a median follow-up of 28.5 months (range: 8–58 months), where patients underwent flap reconstruction with or without adjuvant therapy at our institution. Owing to the small cohort size, analyses were primarily descriptive. Continuous variables were summarized as the median, interquartile range, and range, whereas categorical variables were reported as frequencies and percentages. Exact 95% confidence intervals were calculated for selected proportions.

**Results:**

The cohort comprised 7 male (46.7%) and 8 female (53.3%) patients, with a median age of 65.0 years (IQR, 52.0–76.8 years; range, 31–86 years). All patients achieved microscopic negative margins (R0 resection), and 10 (66.7%) received adjuvant therapy. The median flap surface area was 120 cm² (IQR, 80–171 cm^2^; range, 42–240 cm^2^). Five patients developed postoperative complications—including partial flap necrosis, delayed donor- or graft-site healing, infected sinus tract formation, and hematoma—yielding a complication rate of 33.3% (exact 95% CI, 11.8%–61.6%). Complete wound closure was eventually achieved in all cases. Over the follow-up period, five deaths (four disease-related) and four cases of disease progression were documented.

**Conclusion:**

In this single-center retrospective series, flap-based reconstruction was a feasible limb-salvage strategy after lower extremity STS resection, providing reliable coverage for complex defects. Although postoperative complications occurred in some patients, flap reconstruction remained an important component of multidisciplinary limb-preservation treatment, particularly when combined with careful perioperative management.

## 1 Introduction

Soft tissue sarcomas (STS) comprise a diverse group of malignant tumors that can develop at virtually any anatomical site, though they most commonly affect the lower extremities (1). Historically, amputation was the treatment of choice; however, a pivotal study by Rosenberg et al. in 1982 found no significant difference in disease-free or overall survival between amputation and limb-sparing surgery (2, 3). This crucial finding has established limb-sparing surgery as the standard approach for STS management. Due to the deep-seated nature of STS near vital structures and the high recurrence risk, guidelines recommend wide resection with negative margins, which may result in complex soft tissue defects requiring advanced reconstructive strategies.

In recent years, pedicled and free tissue transfer techniques have become increasingly common for limb salvage following resection of lower extremity STS (4). Up to 85% of patients undergoing resection of thigh STS require some form of wound reconstruction, including skin grafts and flap procedures (5, 6). The need for flap coverage is even greater in areas such as the calf and foot, where soft tissue is scarce. Although the use of pedicled or free flaps increases the technical complexity of STS surgery, previous reports suggest that appropriately selected flap reconstruction can provide reliable soft tissue coverage without increasing postoperative complication rates compared with primary wound closure. Furthermore, contemporary reconstructive strategies increasingly emphasize tailoring flap selection to defect characteristics, anatomical location, and tissue requirements rather than relying on a single preferred technique (7, 8). The surgical management of lower extremity STSs may be further complicated by tumor involvement or intraoperative exposure of critical neurovascular structures. In cases requiring reconstruction of major vascular axes, oncovascular reconstruction represents an integral yet technically demanding component of limb-salvage surgery. However, optimal decision-making and technical strategies for oncovascular reconstruction remain controversial, with current evidence largely derived from limited high-level studies, as highlighted by recent literature on femoral-axis reconstruction in thigh sarcomas (9).

Although flap-based repair is fundamental for soft tissue reconstruction, the clinical benefit of adjuvant therapy in STS management remains controversial. Currently, the impact of such adjuvant regimens on flap reconstruction after extensive lower extremity STS resection—specifically concerning wound healing, recurrence, and complications—remains unaddressed. Addressing this critical gap and the absence of consensus guidelines, we retrospectively reviewed our institutional cohort to characterize postoperative complications and oncologic outcomes in patients undergoing combined flap reconstruction and adjuvant therapy, providing further insights into the clinical outcomes and reconstructive considerations of this integrated treatment strategy.

## 2 Patients and methods

### 2.1 Patients

This retrospective analysis examined 15 cases of lower extremity STS treated at our center from April 2020 to December 2024. The inclusion criteria were as follows: (1) age ≥18 years; (2) lower extremity soft tissue defects; and (3) no relevant vascular disease on preoperative vascular examination. The exclusion criteria were as follows: (1) age <18 years; (2) poor nutritional status and inability to tolerate surgery; and (3) presence of peripheral vascular disease or a related surgical history. Preoperative vascular status was systematically evaluated in all patients using lower extremity vascular ultrasonography; computed tomography angiography (CTA) was additionally performed for tumors closely adjacent to or encasing major neurovascular bundles, as well as for cases scheduled for free flap reconstruction. Patients requiring major oncovascular resection or vascular reconstruction were not explicitly excluded by protocol, but were absent from this cohort as major vascular axes were successfully preserved in all cases. Data from epidemiological, radiographic, and histological assessments, as well as details on surgical techniques, local recurrence, distant metastasis, and complications, were reviewed. Imaging studies conducted at the time of presentation, including radiographs and MRIs, were also included. Diagnoses were confirmed through histological and immunohistochemical examination by expert pathologists. None of the patients in this cohort received preoperative or postoperative radiotherapy. Postoperative adjuvant radiotherapy was omitted following multidisciplinary tumor board evaluation, considering the increased risk of acute microvascular/wound complications during early flap integration and the loss of standard oncologic timing if delayed beyond flap maturation (>3 months).

Patient demographics and tumor characteristics, including location, size, histopathology, and surgical details, were obtained from electronic medical records. Tumor size was further stratified according to the size-based staging criteria of the American Joint Committee on Cancer (AJCC). Given the limited sample size of our cohort, patients were dichotomized into two categories (≤10 cm and >10 cm) to facilitate an exploratory subgroup analysis. The location and selection of flaps for lower limb STS are summarized in Table 1.

**Table 1:** Patient demographics.

Demographic	*n*
Total number of patients	15
Males	7
Females	8
Age(year)	
>60	9
≤60	6
Current tobacco use	2
Diabetes	2
Hypertension	5

### 2.2 Surgery

#### 2.2.1 Tumor removal

This was a retrospective study of consecutive cases performed by the same combination of orthopedic oncology surgeon.

**Figure 1:**
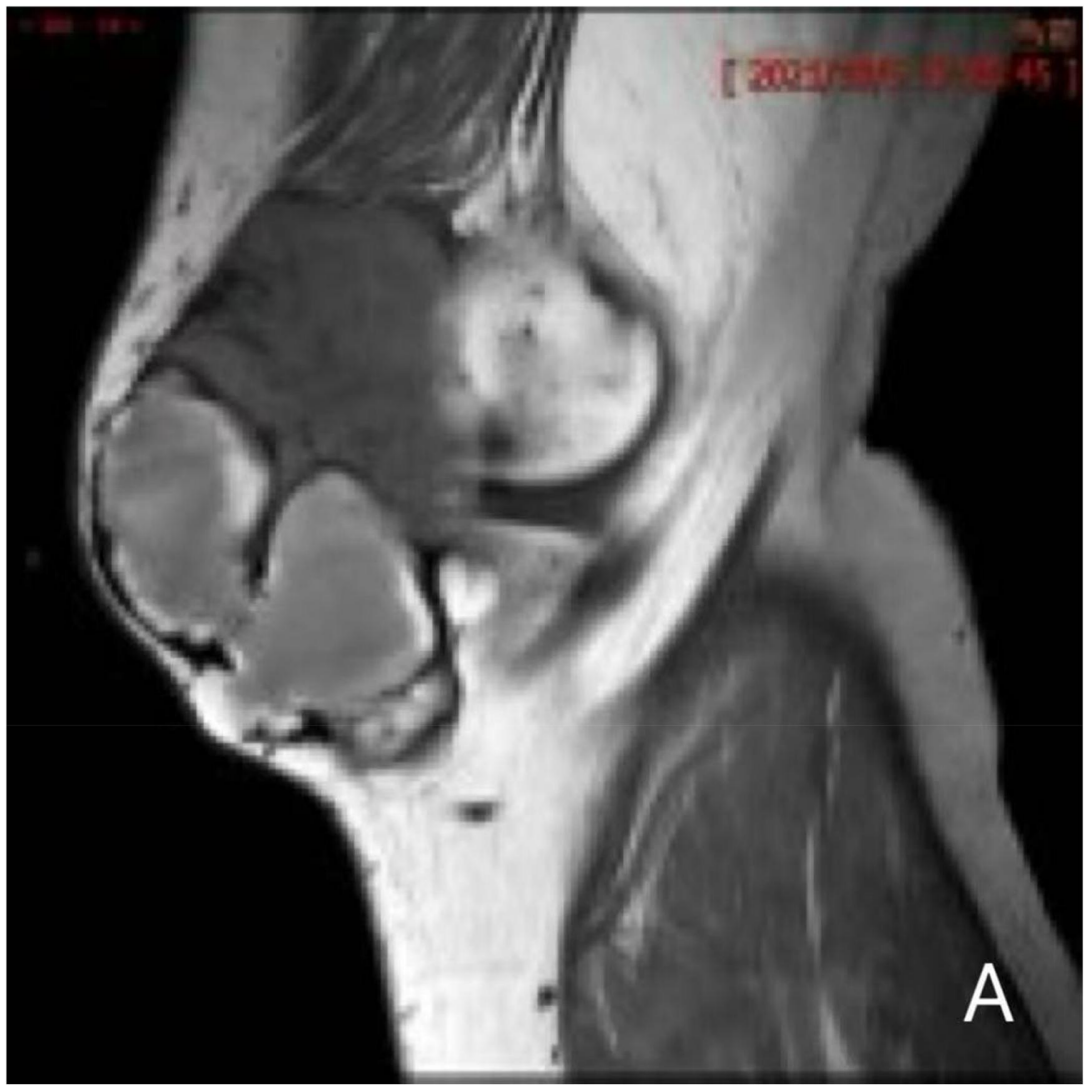
Preoperative MRI showed intra-articular extension of the lesion into the patellofemoral joint cavity.

**Figure 2:**
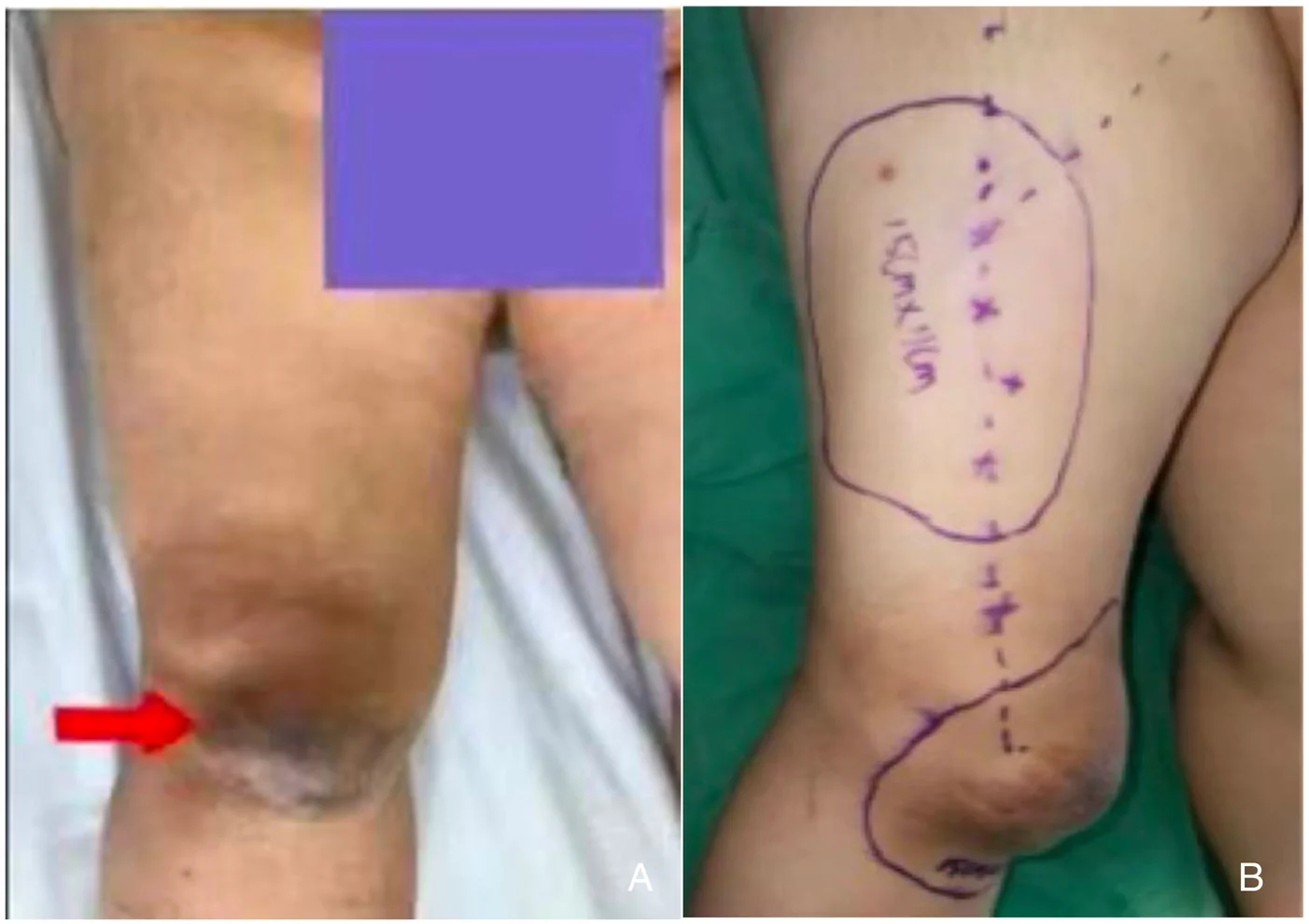
**(A,B)** preoperative surgical planning. Preoperative MRI demonstrated a right knee joint tumor measuring 10 cm × 8 cm × 6 cm **(A)**, based on which the flap type and preoperative dimensions were delineated **(B)**.

All patients were managed in accordance with the NCCN Guidelines for STS (Version 1.2024). Resection margins of 1.0–3.0 cm were planned based on tumor stage and MRI findings(Figures 1, 2A,B). For patients with prior excisions elsewhere, a wide elliptical incision extending 1.0–2.0 cm beyond the previous scar was used to remove potentially contaminated tissue. Intraoperative frozen-section pathology was routinely performed on peripheral and deep margins to verify negative surgical margins, with further margin enlargement executed as needed to guarantee an R0 resection. Following tumor removal, adjacent soft tissue was mobilized, and deeper layers were sutured to minimize defects while preserving blood supply.

#### 2.2.2 Flap reconstruction

**Figure 3:**
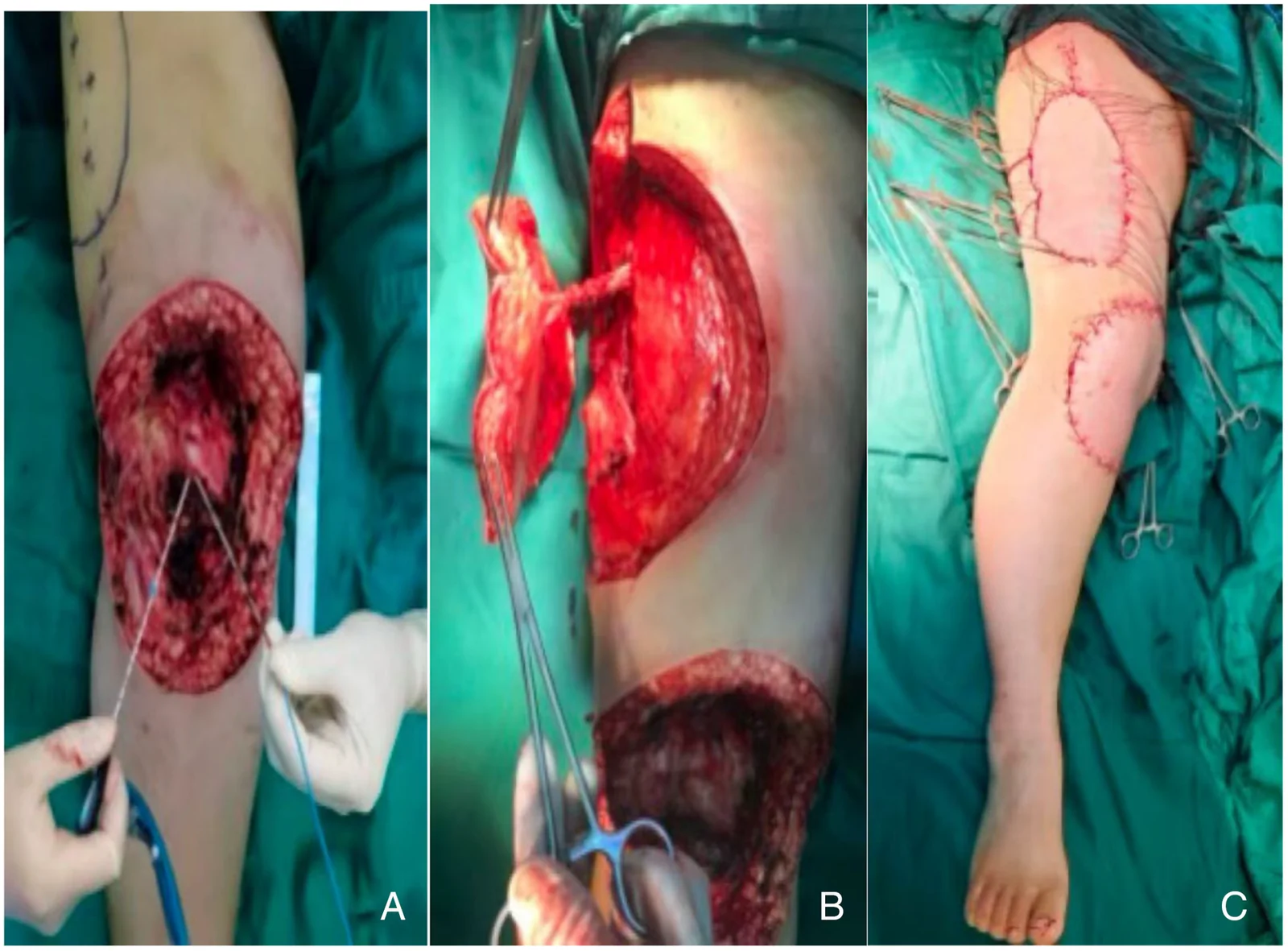
**(A–C)** intraoperative flap design and elevation. A retrograde anterolateral thigh (ALT) flap was meticulously designed, with its pedicle clearly isolated and visualized. **(C)** Soft tissue reconstruction and donor-site closure. The flap was rotated to cover the defect, and the residual donor site was closed using a split-thickness skin graft harvested from the adjacent thigh.

All reconstructive procedures were performed at the index surgery. In free flap procedures, incisions were made along preoperative markings according to reconstructive requirements, followed by meticulous exploration of the target recipient and donor arteries and veins. Upon confirming favorable vascular conditions, the vascular pedicle was carefully dissected to isolate the perforating and main vessels while meticulously preserving perforator integrity. Following complete flap harvesting, the pedicle was trimmed to the required length, and the neurovascular structures were prepared for anastomosis. The flap was then transferred to cover the defect remaining after soft tissue tumor resection. Microvascular arterial and venous anastomoses were performed under an operating microscope. Excellent microvascular patency was confirmed, with the flap demonstrating vibrant tissue perfusion and a healthy pink appearance. Pedicled or local flaps followed a similar sequence: perforators were dissected to the necessary length, the flap was elevated to verify adequate vascularity, and it was rotated to cover the defect while preserving pedicle integrity (Figures 3A–C).

Adjunct wound-support strategies (including NPWT/VAC, dermal substitutes, split-thickness skin grafting, or delayed closure) as well as bone reconstruction or joint/tendon resections were not required in any patient, as well-vascularized flaps provided complete full-thickness defect coverage in a primary setting.

#### 2.2.3 Post-operative care

During the post-operative phase, it is essential to elevate the lower extremity to minimize swelling. Additionally, maintaining warmth, avoiding smoking, and administering symptomatic treatments, such as antibiotics, anticoagulants, and vasodilators, are critical. Patients with compromised blood vessels, vascular crises, or hematomas should receive prompt intervention, with reoperation considered if necessary. The drain is removed three days post-surgery, and adjuvant therapy is initiated once the postoperative incision has healed.

### 2.3 Adjuvant therapy

Neoadjuvant therapy was defined as systemic therapy, including chemotherapy, targeted therapy, or immunotherapy, administered before surgery. Adjuvant therapy was defined as systemic therapy administered after surgery. Perioperative treatment was defined as systemic therapy administered either before or after surgery. In our cohort, adjuvant radiotherapy was not administered to avoid early radiation damage to the transferred flap microvasculature during its critical 3-month revascularization phase, as well as the loss of optimal oncologic efficacy associated with excessively delayed radiation.

Regarding adjuvant modalities, immunotherapy exclusively involved pembrolizumab (a PD-1 inhibitor), whereas neither PD-L1 nor CTLA-4 inhibitors were administered. Targeted therapy was restricted to anti-angiogenic agents, specifically everolimus and anlotinib. The primary cytotoxic chemotherapy consisted of the ifosfamide and adriamycin (AI) regimen, which was also utilized in combination therapy (e.g., anlotinib plus AI). The therapeutic distribution among the 15 patients was as follows: immunotherapy (*n* = 3), targeted therapy (*n* = 3), combination therapy (*n* = 2), chemotherapy alone (*n* = 2), and no adjuvant therapy (*n* = 5). All patients in the immunotherapy group completed at least two full cycles of the PD-1 inhibitor, and those undergoing chemotherapy received their scheduled cycles both pre- and postoperatively.

### 2.4 Study endpoints and outcome assessment

The primary endpoint of this study was flap-related outcomes following soft tissue reconstruction, specifically flap survival, partial flap necrosis, and total flap necrosis. Flap survival was defined as complete flap viability, achieving successful defect coverage without surgical re-intervention. Partial flap necrosis was defined as localized tissue loss managed conservatively or via limited debridement, whereas total flap necrosis was defined as complete loss of flap viability necessitating flap excision or secondary reconstruction.

Secondary endpoints included postoperative wound complications (surgical site infection, wound dehiscence, hematoma, and delayed healing) and time to complete wound healing. Delay to systemic therapy was defined as the postponement of scheduled postoperative chemotherapy, immunotherapy, or targeted therapy attributable to reconstruction-related complications.

Oncologic outcomes comprised local recurrence, distant metastasis, overall survival (OS), and disease-specific survival (DSS).

### 2.5 Statistical analysis

Because this study included only 15 patients and a limited number of postoperative and oncologic events, the statistical analysis was primarily descriptive. Continuous variables were summarized as the median, interquartile range (IQR), and range, and the number of patients with available data was reported for each variable. Categorical variables were presented as frequencies and percentages, with the corresponding denominator specified when data were incomplete.

For selected clinically relevant proportions, including the rates of postoperative complications, disease progression, mortality, and flap type, two-sided exact 95% confidence intervals were calculated using the Clopper–Pearson method. Missing data were not imputed, and all summaries were based on available observations.

## 3 Result

This retrospective case series included 15 patients, comprising 7 males and 8 females, who underwent immediate flap reconstruction following lower-extremity STS resection. The median age of the cohort was 65.0 years (range, 31–86 years). Over a median follow-up period of 28.5 months (range, 8–58 months), a total of five deaths were recorded.

### 3.1 Patient characteristics and surgical outcomes

As summarized in Table 1, the cohort consisted of 15 patients with lower extremity STS (7 males [46.7%], 8 females [53.3%], and the median age was 65.0 years (IQR, 52.0–76.8 years; range, 31–86 years). Nine patients (60.0%) were aged >60 years, and six (40.0%) were aged ≤60 years. Preoperative comorbidities and personal history included hypertension in five patients (33.3%), diabetes in two (13.3%), and a history of active or former smoking in two (13.3%). As shown in Table 2, the most common tumor location was around the knee (7 patients, 46.6%), followed by the hip (3 patients, 20.0%), calf (2patients, 13.3%), foot (2 patients, 13.3%), and proximal thigh (1 patient, 6.7%). Among the 5 patients with a tumor diameter greater than 10 cm, three underwent reconstruction using free flap transplantation, while the remaining 2 patients, alongside all 10 patients with a tumor diameter less than or equal to 10 cm, were treated with pedicled flap repairs. To repair these post-oncological defects, a total of 15 flaps were successfully transferred, including three free flaps (one free latissimus dorsi flap and two free anterolateral thigh [ALT] flaps) and 12 pedicled flaps (7 ALT flaps, 2 lateral gastrocnemius muscle flaps, 1 medial gastrocnemius muscle flap, 1 posterior tibial artery perforator flap, and 1 sural neurocutaneous flap). All patients initially presented with a localized mass; 6 (40.0%) reported symptoms such as ulcers (3), venous dilation (2), and numbness (1). Undifferentiated pleomorphic sarcoma was the most common histological subtype, accounting for 9 cases (60.0%), followed by myxofibrosarcoma in 3 cases (20.0%), synovial sarcoma in 2 cases (13.3%), and inflammatory myofibroblastic sarcoma in 1 case (6.7%). According to the 8th edition of the AJCC staging system, 2 patients (13.3%) were classified as stage II, 5 (33.3%) as stage IIIA, and 8 (53.3%) as stage IIIB. Perioperative treatments included doxorubicin plus everolimus (2 patients), neoadjuvant and adjuvant chemotherapy (2), targeted therapy with anlotinib plus everolimus (3), and pembrolizumab immunotherapy (3), while 5 patients (33.3%) received no adjuvant therapy. Post-operative adjuvant chemotherapy was typically initiated 3 weeks after the reconstructive surgery. The exact timing was determined through multidisciplinary team (MDT) consultations and was strictly contingent upon complete wound healing and the stabilization of the transferred flaps, ensuring that systemic therapy would not compromise flap viability or wound integrity.

**Table 2:** Tumor and treatment characteristics.

Demographic	*n*
Tumor location	
Hip	3
Thigh	1
Knee	7
Calf	2
Foot	2
Side	
Left	11
Right	4
Tumor size(cm)	
>10	5
≤10	10
Histology	
Undifferentiated Pleomorphic Sarcoma	9
Myxofibrosarcoma	3
Synovial sarcoma	2
Inflammatory myofibroblastic sarcoma	1
Tumor stage	
AJCC Ⅱ	2
AJCC ⅢA	5
AJCC ⅢB	8
Margin status	
R0	15
R1	0
Adjuvant therapy	
Chemotherapy	2
Immunotherapy	3
Targeted therapy	3
Combined therapy	2
Flap choice	
Pedicled flap	12
Anterolateral thigh flap	7
Lateral gastrocnemius muscle flaps	2
Medial gastrocnemius muscle flap	1
Posterior tibial artery perforator flap	1
Sural neurocutaneous flap	1
Free flap	3
Free latissimus dorsi flap	1
Free anterolateral thigh flap	2
Flap Area(cm^2^)	
>100	10
≤100	5

Numeric intraoperative blood-loss data were available for 13 patients, with a median blood loss of 400 mL (IQR, 300–450 mL; range, 100–1,000 mL). Because of the small number of patients, flap size, blood loss, patient age, flap type, and systemic treatment were not assessed using regression models or formal between-group hypothesis testing.

### 3.2 Tumor and oncology survival

All patients underwent preoperative MRI, which identified a soft tissue mass with indistinct margins, and the median tumor volume was 149.1 cm^3^ (IQR, 68.0–269.1 cm³; range, 4.5–597.6 cm^3^). In two cases (13.3%), a septum was observed within the tumor. Two patients (13.3%) had tumors invading the skin, while two patients (13.3%) exhibited abundant hematopoiesis within the STS. Four patients (26.7%) had sarcomas involving peripheral muscles, and another four (26.7%) had tumors infiltrating bones and joints. All patients underwent preoperative chest CT to rule out distant metastases, and three patients (20.0%) also had a PET-CT scan.

At the last follow-up, 5 of 15 patients had died, corresponding to an all-cause mortality proportion of 33.3% (exact 95% CI, 11.8%–61.6%). Four deaths were attributed to disease progression, whereas one patient died from an unrelated cause. Documented disease progression occurred in 4 patients (26.7%; exact 95% CI, 7.8%–55.1%), including local recurrence and distant metastatic progression.

Given the limited number of deaths and progression events, median survival, subgroup survival comparisons, Cox regression estimates, and adjusted prognostic analyses were not reported.

### 3.3 Flap repair and complications

Regarding anatomical distribution and lateralization, 2 reconstructions (13.3%) were performed in the calf, 7 (46.7%) were located around the knee, and the remaining 6 cases were evenly distributed across the foot (*n* = 2,13.3%), thigh (*n* = 2,13.3%), and hip (*n* = 2,13.3%). Notably, a significant predominance of left-sided reconstructions was observed, with 11 patients (73.3%) presenting with left-sided defects compared to 4 patients (26.7%) with right-sided defects.

**Figure 4:**
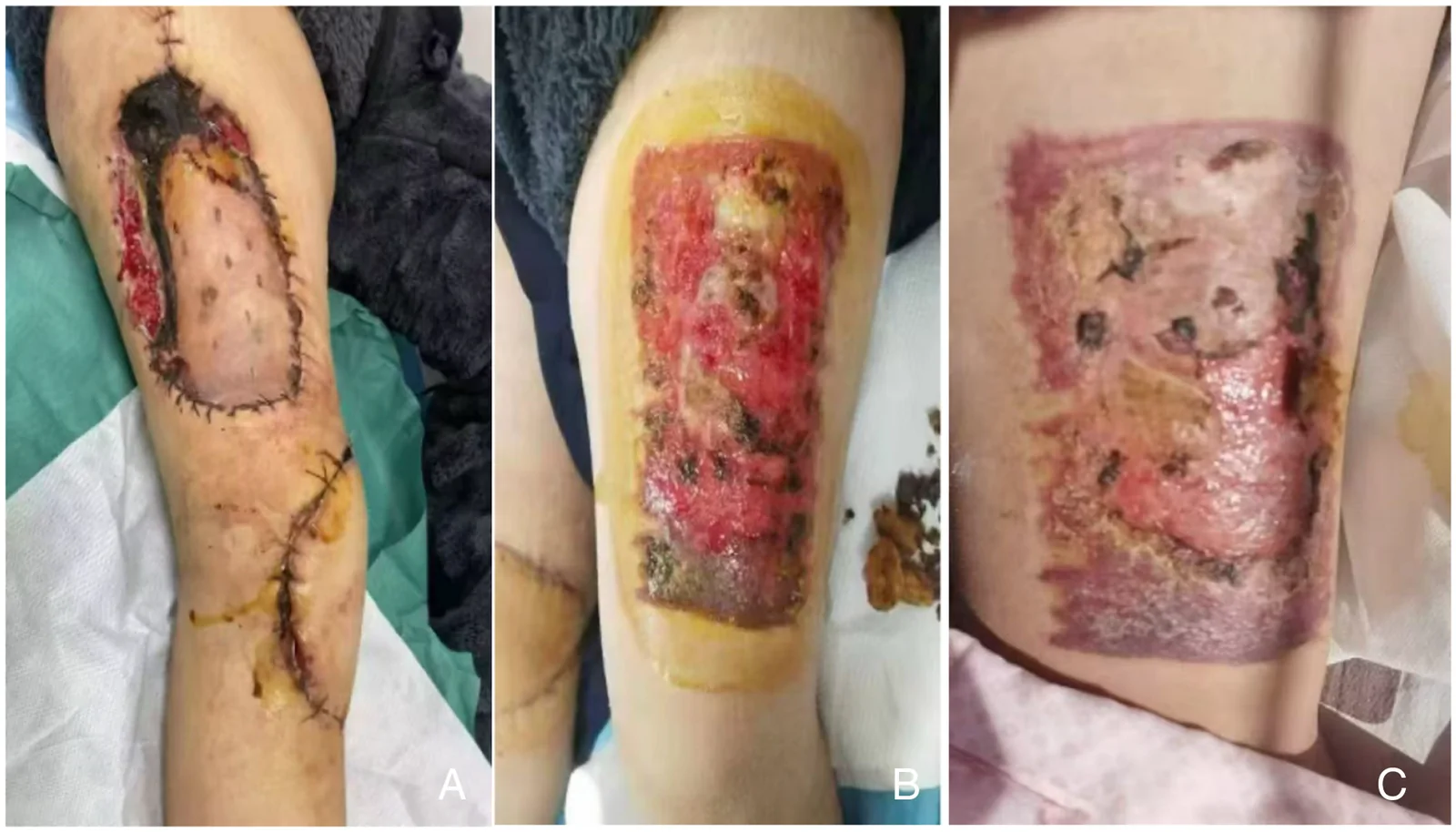
**(A–C)** early postoperative wound complications, showing partial necrosis of the skin graft with localized wound exposure and delayed donor-site healing with persistent exudation. Complete donor-site healing with residual scarring achieved after meticulous dressing changes.

**Figure 5:**
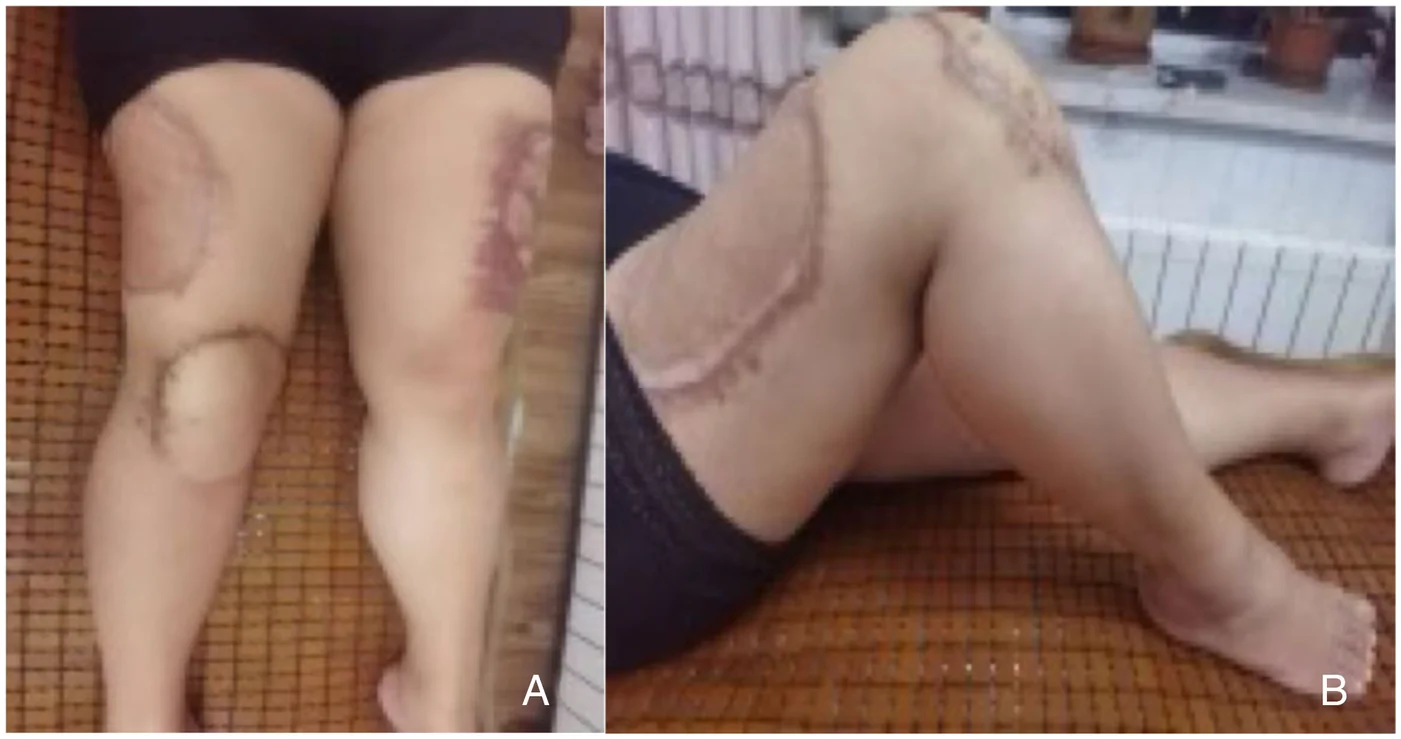
**(A,B)** At the 2-year follow-up, the transferred ALT flap showed complete survival, alongside stable donor-site skin graft healing.

Flap dimensions were available for all 15 patients. The median flap surface area was 120 cm^2^ (IQR, 80–171 cm^2^; range, 42–240 cm^2^). During the postoperative surveillance window, 5 patients (33.3%) experienced surgical complications at various chronological checkpoints, including two cases of partial flap necrosis, one case of donor site/graft site nonhealing, one instance of infected sinus tract formation, and one hematoma. Following the administration of indicated systemic adjuvant medications and subsequent targeted wound care, all 5 cases ultimately exhibited delayed healing but achieved complete closure (Figures 4A–C, 5A,B). A granular analysis of the necrosis cohort revealed that 2 patients had been undergoing targeted therapy and chemotherapy, respectively, with 1 patient concurrently demonstrating delayed healing at the adjacent split-thickness skin graft donor site. The remaining instance of partial flap necrosis occurred in a patient managed with multi-agent combination chemotherapy. Because only five complication events occurred, the study did not attempt to identify independent predictors of complications or determine a clinically meaningful flap-area threshold. In particular, the observed occurrence of complications in patients with larger flaps should be regarded as a descriptive observation rather than evidence of a statistically established association.

## 4 Discussion

The primary objective of this study was to directly delineate the postoperative complication profiles and clinical outcomes associated with flap-based reconstruction following extended resection of lower extremity STSs, specifically within the context of perioperative adjuvant therapy. Surgical resection of STS is generally linked to a high incidence of postoperative complications, especially in the lower extremities (10, 11). Optimal wound healing in this cohort is frequently compromised by a multifaceted interplay of both patient-centric and treatment-related variables, encompassing the administration of systemic adjuvant regimens, advanced age, substantial tumor volume, and metabolic or lifestyle comorbidities such as diabetes mellitus and tobacco use. Pinpointing these critical risk factors holds paramount clinical significance; persistent wound or flap complications not only cause surgical distress but can also fatally delay the initiation of mandatory adjuvant therapies, ultimately jeopardizing overall oncologic survival.

Reconstruction of soft tissue defects following resection of lower extremity STS remains a complex and technically challenging aspect of multidisciplinary care. Both pedicled and free flap techniques are widely used, with current evidence indicating comparable rates of postoperative complications between the two approaches when appropriately selected (12, 13). The proximal thigh is the most common site of sarcoma in lower extremity STS, accounting for 42% of cases (14, 15). However, in our study, lower extremity STS predominantly occurred around the knee joint (16, 17), particularly in elderly patients with undifferentiated pleomorphic sarcoma (UPS) (18, 19), as confirmed by Penna et al.

Reconstruction following oncologic resection should be individualized according to defect characteristics, anatomical location, tissue requirements, and the anticipated need for subsequent therapies. Previous studies have proposed reconstructive algorithms based on these principles, emphasizing that flap selection should be tailored rather than determined by a single preferred technique (16), Brunetti et al. reported an algorithm for thigh reconstruction in oncologic patients, highlighting the importance of defect size, depth, irradiation status, and reconstructive objectives in guiding the selection of local, pedicled, or free tissue transfer (6, 20). In our cohort, flap reconstruction followed a defect-oriented strategy, with flap selection determined by defect dimensions, anatomical location, dead-space management requirements, and recipient vessel availability. Pedicled perforator flaps were the most frequently used approach, particularly in cases requiring reliable and durable soft tissue coverage. Among these, the perforator-based anterolateral thigh (ALT) flap was commonly selected because of its consistent vascular anatomy, reliable perfusion, versatile tissue composition, and adaptability in flap design with minimal donor-site morbidity. The ALT flap was primarily used for medium-to-large defects involving the anterior and lateral thigh or upper leg. For lower-third thigh or perigenicular defects where the antegrade pedicle length was insufficient, reverse-flow ALT flap harvest based on anastomotic connections between the descending branch of the lateral circumflex femoral artery (LCFA) and the lateral superior genicular artery was utilized to extend the reconstructive reach (5, 21). For deep, three-dimensional or infected defects following radical sarcoma resection, muscle or myocutaneous flaps (e.g., latissimus dorsi or gastrocnemius flaps) were selected to provide substantial tissue bulk for obliterating large dead spaces and protecting vital neurovascular structures or orthopedic hardware. Specifically, pedicled medial or lateral gastrocnemius muscle/myocutaneous flaps were utilized as localized, highly reliable options for soft tissue coverage around the distal thigh and peri-knee region. Conversely, in cases with extensive defects exceeding local options or where recipient site contamination precluded local flap rotation, free microvascular tissue transfer (e.g., free ALT or free latissimus dorsi flap) was performed, leveraging distant recipient vessels (such as the anterior or posterior tibial vessels) outside the zone of radiation or tumor resection (5, 22). This tailored strategy ensured durable, well-vascularized tissue coverage even in biologically and surgically challenging environments.

In planning flap reconstruction for lower extremity STS defects, deep wound edges were routinely retracted and sutured to assess tissue mobility, potentially reducing defect size. Flaps were designed to match the original defect dimensions, ensuring tension-free coverage while preserving vascular integrity. In 13 of 15 patients, the flap size matched the defect. In two cases—one plantar and one anterior tibial with infection—the flap size exceeded the defect by approximately 0.5 cm to ensure adequate perfusion and minimize tension. This approach contrasts with post-traumatic reconstructions, where tissue damage limits elasticity and mobility. In such cases, flaps are typically designed to exceed the defect size by 1–2 cm to prevent excessive tension (23), which is a known risk factor for ischemia and partial flap necrosis. In contrast, sarcoma resections generally involve healthy tissue with intact vessels, permitting precise flap shaping and tension-free closure without the need for excessive sizing. This approach may facilitate reliable soft tissue coverage and contribute to favorable reconstructive results in lower extremity reconstruction.

In our follow-up, two of the 15 patients who underwent flap reconstruction experienced local recurrence, and two patients died from distant metastases, highlighting the aggressive nature of high-grade STS. One of the patients who developed local recurrence had undifferentiated pleomorphic sarcoma (UPS) and a 60-year smoking history. Among the two patients who developed distant metastases, one had synovial sarcoma, and the other had UPS. Notably, all four of these patients had undergone pedicled flap reconstruction for post-resection defects and were classified as AJCC stage III at the time of treatment. These findings suggest that advanced tumor stage and aggressive histologic subtype may have contributed substantially to disease progression in this subgroup, while long-term smoking exposure may also have played a role in the unfavorable oncologic outcome observed in the patient with local recurrence. Among patients who received adjuvant therapy, one patient showed a partial response to neoadjuvant chemotherapy, with reduced necrosis, edema, and tumor size. Two patients received neoadjuvant immunotherapy for large-volume tumors, leading to a 20% reduction in tumor size and improved tumor margin demarcation (24). Additionally, targeted therapies have been observed to be associated with extended progression-free survival (PFS) in patients with advanced disease (25). While these adjuvant modalities may offer potential clinical benefits, their presence introduces complex considerations regarding wound healing, particularly in cases involving flap reconstruction (26, 27). Cytotoxic agents, such as anthracyclines and taxanes, can induce myelosuppression and neutropenia, which might alter the early inflammatory phases of tissue repair. Targeted agents (e.g., pazopanib) could theoretically influence flap perfusion through endothelial cell inhibition, whereas immune checkpoint inhibitors might elicit immune-mediated tissue responses that could potentially influence local inflammation or fibrosis. Consequently, while adjuvant therapies remain an important component of comprehensive oncologic management (24–27), they may contribute to a challenging local tissue microenvironment during wound healing. Achieving a delicate balance between thorough oncologic resection and reliable soft-tissue reconstruction remains essential. Furthermore, evaluating surgical margin status following neoadjuvant targeted therapy or immunotherapy presents unique challenges due to therapy-associated histopathological alterations.

The clinical prognosis of lower extremity STS is shaped by a multifaceted interplay of variables, among which chronological age and specific histologic subtype stand out as paramount determinants. Evidenced by robust, large-scale multicenter data, individuals aged ≥75 years exhibit a strikingly elevated 5-year local recurrence rate of 12.9% compared to a mere 7.2% in their counterparts under 30 years of age, a disparity that is consistently mirrored in their respective distant metastasis rates (33.9% vs. 17.5%) (28). Similarly, distant metastasis rates were 33.9% and 17.5%, respectively. In the present cohort, UPS (29, 30) were the most common tumor types, and it is associated with a high degree of malignancy as well as an increased risk of recurrence and distant metastasis. Characterized by aggressive biological behavior and profound chromosomal chaos, UPS is inherently predisposed to early recurrence and widespread hematogenous dissemination. Notably, the marked propensity of UPS to afflict the elderly population may be rooted in age-dependent biological transitions. These include accelerated genomic instability, systemic immunosenescence, and a progressively permissive tumor microenvironment (TME) that collectively orchestrate and fuel tumor virulence. Regrettably, despite recent therapeutic breakthroughs in precision medicine, a significant subset of patients inevitably succumbs to either primary or acquired chemoresistance, ultimately culminating in suboptimal long-term oncologic outcomes.

This study has several limitations. First, it is a retrospective, non-randomized, single-institution analysis with a relatively small sample size, which may limit the generalizability of the findings. Second, because STS comprises a highly heterogeneous group of malignancies with distinct histological subtypes, the limited number of cases within each subtype precluded subtype-specific analysis, thereby restricting the interpretability of the data. Third, perioperative radiotherapy was not administered in this cohort due to concerns regarding early microvascular-compromised flap survival and the loss of standard oncologic timing if delayed beyond flap maturation; while this minimized radiation-induced wound complications, it may limit the applicability of our findings to patients receiving combined radiotherapeutic protocols. Fourth, the short follow-up period and the lack of objective evaluation limited a comprehensive assessment of long-term outcomes and potential complications. In addition, active smoking and diabetes were present in a subset of patients and may have acted as potential confounding factors affecting flap-related outcomes; however, given the limited sample size and the small number of complication events, robust adjustment for these variables was not feasible. Future prospective studies with larger cohorts and longer follow-up are warranted to validate these preliminary findings.

## 5 Conclusion

In conclusion, current evidence suggests that optimizing lower extremity STS management relies on balancing radical tumor resection with appropriate soft tissue reconstruction. Free or pedicled perforator flaps, including ALT and gastrocnemius designs, appear to provide an effective option for defect coverage. Although systemic adjuvant treatments may introduce additional wound-healing risks, their integration should be carefully considered to help balance oncological control with functional limb preservation.

## Data Availability

The original contributions presented in the study are included in the article/Supplementary Material, further inquiries can be directed to the corresponding author/s.
